# Assessing the students’ evaluations of educational quality (SEEQ) questionnaire in Oman higher education

**DOI:** 10.12688/f1000research.157354.1

**Published:** 2024-11-06

**Authors:** Muna Al Kalbani

**Affiliations:** 1 Computing, Muscat College, Ruwi, Muscat Governorate, Oman

**Keywords:** SEEQ, Student Evaluations of Teaching (SET), validity, reliability, teaching

## Abstract

**Background:**

One of the most popular tools that is used to evaluate education quality is the Student Evaluation of Education Quality (SEEQ). This local data study examined the SEEQ’s psychometric properties in Omani education, specifically its validity and reliability.

**Methods:**

This was quantitative research to assess educational quality from the students’ viewpoint. The study was conducted at Muscat College from January to March 2024.Simple randomization implemented at the course level helped to choose the participants. All students registered in the specified courses were asked to take part after a random sample of the courses Muscat College offers throughout the study term was chosen. Five hundred fifty students completed the survey. AMOS was used to conduct confirmatory factor analysis (CFA), and PSS was used to evaluate the internal consistency (IBM Corp., 2024a, 2024b).

**Results:**

CFA of the SEEQ indicated that the eight -factor solution best fit the data. Results also showed that the SEEQ has overall good-to-excellent reliability. The adapted scale was psychometrically sound, proving SEEQ’s applicability

**Conclusions:**

This study proposes using SEEQ to measure Oman’s education quality.

## 1. Introduction

Course and program evaluation is a quality control process performed internally by all colleges and universities. Student evaluations of teaching (SET) are performed by delivering questionnaires that employ rating scales, such as the Likert scale. These scales enable students to indicate their degree of either agreeing or disagreeing with certain statements. While SETs were originally designed for evaluating courses and programs, they have since been used to assess teaching effectiveness. Various teaching aspects, including group interaction, preparation and organization, and student feedback, are often evaluated when evaluating the effectiveness of a teacher ( Constantinou & Wijnen-Meijer, 2022). The data collected through SETs can provide valuable insights into the need for modifying the instructional material or teaching approach used in a specific course. SETs have also been utilized to assist make valuable decisions on the development of academic careers. Prospective faculty members are frequently asked by institutions to supply their Student Evaluation of Teaching (SET) outcomes from previous institutions as a requirement of their application for a new position. This requirement greatly impacts the professional progression of faculty members, both within their current institution and when seeking job opportunities outside ( Constantinou & Wijnen-Meijer, 2022).

Evaluation tools provide a way for individuals to enhance and assess the standard of education. In these days, instruments are frequently employed in educational research, especially when students assume the role of assessors. Marsh’s (1982) The SEEQ is one of the most popular SET tools used in the education sector. It has been employed in many locations, including higher learning institutions in multiple nations ( Grammatikopoulos et al., 2015). SEEQ has been developed with a comprehensive consideration of the various facets of teaching and learning, enabling its applicability across all academic disciplines. It is widely used worldwide and considered one of the most accurate and useful measures to measure teaching effectiveness ( Al-Muslim & Arifin, 2015). Marsh (1982) discovered that the SEEQ scale displayed internal consistency within the range of 0.88 to 0.97 in the United States. In Australia, the utilization of SEEQ resulted in an internal consistency level of 0.89, as reported by Marsh and Roche in 1993. In a study conducted in Oman in 2011, Cronbach’s alpha value of 0.87 was reported by Al-Hinai. SEEQ has shown positive psychometric results across various linguistic and cultural contexts, yet, it has not yet been administered to college students in Oman. Based on the researcher’s knowledge, no previous studies have examined the psychometric properties of the SEEQ questionnaire among colleges and universities students in Oman. This study aims to investigate the psychometric properties of the SEEQ among college students in Oman, so as to fill a research gap. Specifically, the goals of this study are as follows: (a) Evaluate the reliability of the Arabic version of SEEQ, and (b) Find out how many dimensions it has by using confirmatory factor analysis.

## 2. Methods

### 2.1 Study design

The Students’ Evaluations of Educational Quality (SEEQ) Questionnaire ( Marsh & Roche, 1993) is used in this quantitative research to assess educational quality from the students’ viewpoint. The study was conducted at Muscat College from January to March 2024.

Collecting numerical data for statistical analysis is a fundamental aspect of the quantitative technique, facilitating the identification of correlations and patterns within the dataset ( Creswell & Creswell, 2017). The research aimed to contribute to the broader discourse on assessing higher education by gathering data on student evaluations of course quality ( Hancock & Algozzine, 2016).

### 2.2 Sample size calculation and sampling method

A total of 278 students are needed to be surveyed to achieve a 95% confidence interval, a 5% significance level, and 80% power. The participants were selected by a simple randomization process. The participants were chosen through a simple randomization process at the course level. Completion is based upon checking the registration department for a comprehensive list of Muscat College students. Simple randomization implemented at the course level helped to choose the participants. All students registered in the specified courses were asked to take part after a random sample of the courses Muscat College offers throughout the study term was chosen. Five hundred fifty students completed the survey. The response rate was 91.7%, with 550 out of 600 individuals participating.

### 2.3 Participants

In the current study, 550 students from three different departments at Muscat College participated by completing the questionnaires (SEEQ) distributed to them. The participants averaged 20 years old, with a 2.54-year standard deviation.

### 2.4 Ethical approval

The ethical approval was received by Muscat College’s Research Ethical Committee under the reference MC/RC/1/2024, dated January 2024. Prior authorization was obtained before starting the search. Because the study only required completing a non-invasive, anonymous survey (SEEQ) measuring student assessments of educational quality, which had been approved by the research committee, all participants provided oral permission before they were contacted. Participants were told that their participation in this research was founded on the principles of confidentiality and autonomy, and that they might withdraw from the study at any moment.

### 2.5 Data collection procedures

The Students’ Evaluations of Educational Quality (SEEQ) Questionnaire was used for data collecting; the chosen sample of students received paper-based questionnaires. The scheduled hour for the questionnaire administration was set; the teacher stayed outside the classroom to provide an objective environment while students were completing the surveys. Following completion of the surveys, a chosen student collected them, sealed them in an envelope, and handed the envelope to the authors. Students were advised that the teacher would not get any direct comments from the assessments, therefore ensuring the privacy of their answers. Three months, from January 2024 to March 2024, defined the data collecting period. Following the ethical standards set by the college, all completed surveys accompanied with signed informed permission forms.

### 2.6 Measures

The Students’ Evaluations of Educational Quality (SEEQ) questionnaire was the tool used ( Marsh, 1982). The SEEQ consists of thirty items that measure eight dimensions under consideration, one of which measures the subject’s overall evaluation. The SEEQ consists of the following dimensions: breadth of coverage, assessment, assignments, learning value, teacher enthusiasm, organization, group interaction, individual rapport, and overall rating ( Marsh, 1982). Each component is rated on a five-point Likert scale: strongly disagree, disagree, neutral, agree, and strongly agree ( Marsh, 1982). The tool’s developer, Professor Herbert March, was contacted personally and granted permission to be used for study ( Marsh, 1982). The author translated SEEQ into Arabic prior to the study, and the instrument was then translated back into English by a bilingual expert who has experience in this area.

Discrepancies that were identified were rectified in the Omani version of the instrument after the bilingual expert and the authors conducted a comparison between the two editions of the instrument (the original and the re-translated version). Subsequently, a preliminary test was administered to a selected group of students and colleagues to evaluate the reliability of the translated SEEQ. Modifications were implemented to improve the semantic significance of some words in Arabic.

### 2.7 Data analysis

The SEEQ scale was validated using a combination of alternate methods and data analysis. An evaluation of the internal consistency of the scale and its three subscales was conducted using Cronbach’s alpha statistics. The structure of the SEEQ factors was examined using confirmatory factor analyses (CFAs) in AMOS v26, which is a structural equation modeling program. The model was evaluated using fit indices that are often used in the discipline ( Blunch, 2008). The Tucker-Lewis index (TLI), goodness-of-fit index (GFI), comparative fit index (CFI), and Chi-Square (χ ^2^) are among the measures. Fit indices of CFI, GFI, and TLI exceeding 0.90 and RMSEA below 0.08 are generally considered acceptable ( Bentler, 1990).

## 3. Results

### 3.1 Reliability of internal consistency

To determine the internal consistency reliability of each SEEQ scale, Cronbach’s alpha was used. The output was assessed using the corrected item-total correlation or alpha if an item was omitted ( Field, 2013). The reliability (alpha) coefficients for the eight 30-item SEEQ scales are presented in Table 1.

**Table 1.** Cronbach’s Alpha for the scale.

Subscale	No of items	Alpha
learning value	4	0.72
Enthusiasm	4	.77
Organization	4	.72
Group interaction	4	.76 .76
Individual rapport	4	.76
BREADTH	4	.76 .65
Examination	3	.65
ASSIGNMENTS	2	.68
Overall Cronbach’s Alpha for the scale	30	.95

### 3.2 Confirmatory factor analysis

**Figure 1.**
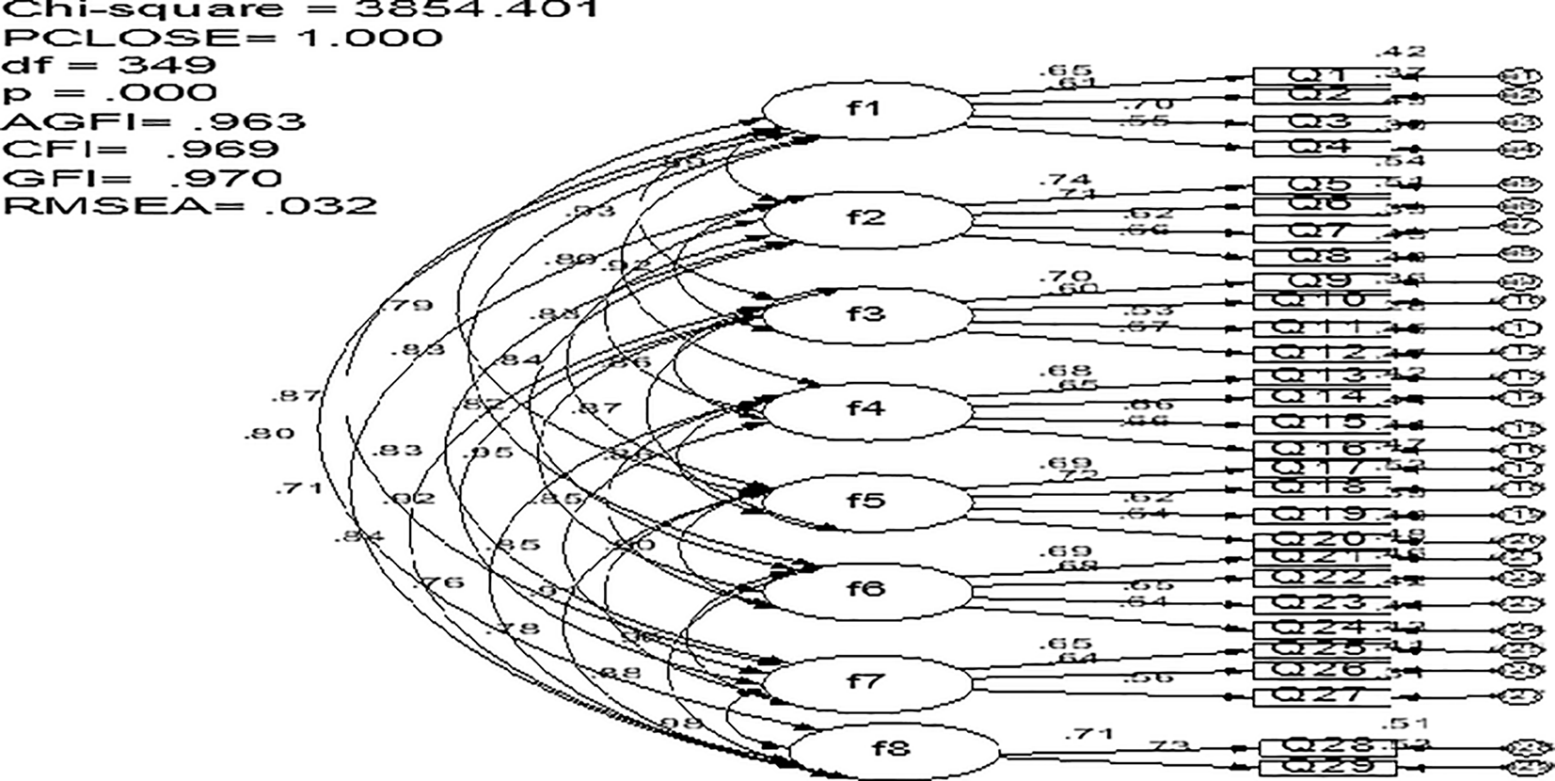
The measurement model for SEEQ.

Confirmatory factor analysis (CFA) is the most suitable technique for cross validating the factor structure of a test, as per Byrne (2001). Using the AMOS version 26.0 statistical tool, a covariance matrix ( Figure 1) was used to evaluate the validity of the item allocation to the eight SEEQ measures ( IBM Corp, 2024a). The eight-factor structure proposed for the original scale has been confirmed by the Arabic version of SEEQ’s acceptable CFA indices ( Figure 1).

## 4. Discussion

The revised CFA model’s goodness-of-fit indices indicate a strong fit to the study data: χ ^2^ (df =349) = 3854.401, CFI = .963, GFI = .970, PCOLOSE = 1.00, and RMSEA = .032. These indices all exceeded thresholds. Cronbach’s Alpha showed 0.935 reliability, demonstrating high internal consistency. The findings support using SEEQ to evaluate educational programs and courses in Oman in the future using similar samples. Because of the small sample size, more research is required to replicate the current study with bigger and more varied samples in order to apply the findings to Oman’s higher education students. This study showed SEEQ’s applicability in Oman’s higher education. Individual Oman instructors may use the SEEQ for gathering student feedback on their instruction and modules.

### Ethical approval

The ethical approval was received by Muscat College’s Research Ethical Committee under the reference MC/RC/1/2024, dated January 2024. Prior authorization was obtained before starting the search. The nature of the research and the little risk involved led to oral consent instead than written permission. Participants in the research had to complete a non-invasive, anonymous survey (Students’ Evaluations of Educational Quality - SEEQ), which only gauges their opinions of educational quality. Written consent was believed to be not required given the low-risk nature of the survey and its anonymous form. The research committee authorized the oral consent procedure as well as the SEEQ tool. All participants provided oral permission before they were contacted. Participants were told that their participation in this research was founded on the principles of confidentiality and autonomy, and that they might withdraw from the study at any moment.

### Consent statement

All participants gave informed consent to be interviewed orally. The college’s ethics committee accepted this method. The source of the informants was who agreed that the data were provided for research purpose and that the data can be published. They also consented that the data were provided for research anonymously without having distorted scientific meaning.

I declare that the data provided is true and can be accounted for.

## Data Availability

Figshare: Data 1_1 - Copy.xls https://doi.org/10.6084/m9.figshare.26494288.v2 ( Al-kalbani, 2024a). This project contains the following underlying data:
• Data 1_1 - Copy.xls Data are available under the terms of the Creative Commons Zero (CC0) All data has been deidentified using the Safe Harbour approach, therefore assuring ethical norms’ conformance. Among the authors who helped to produce this dataset was Muna Alkalbani. Figshare: SEEQ-Arabic version.pdf https://doi.org/10.6084/m9.figshare.27222894.v5 ( Al-kalbani, 2024b). This project contains the following extended:
• ilovepdf_merged (3).pdf Data are available under the terms of the Creative Commons Zero (CC0)

## Software availability statement

IBM SPSS Statistics and IBM AMOS, proprietary program created by IBM Corp., were used in the data analysis for this research. Similar statistical analysis and structural equation modeling may be done using free substitutes such PSPP and the R package lavaan. Lavaan may be accessible via the R environment at https://lavaan.ugent.be/. PSPP is available at [ https://www.gnu.org/software/pspp/].
