# Assessing the students’ evaluations of educational quality (SEEQ) questionnaire in Oman higher education

**DOI:** 10.12688/f1000research.157354.2

**Published:** 2025-02-10

**Authors:** Muna Al Kalbani

**Affiliations:** 1 Computing, Muscat College, Ruwi, Muscat Governorate, Oman

**Keywords:** SEEQ, Student Evaluations of Teaching (SET), validity, reliability, teaching

## Abstract

**Background:**

Student Evaluation of Education Quality (SEEQ) is a widely used tool for assessing educational quality. This local data study examined the SEEQ’s psychometric properties in Omani education, specifically its validity and reliability. This local data study examined the SEEQ’s psychometric properties in Omani education, specifically its validity and reliability.

**Methods:**

This was quantitative research to assess educational quality from the students’ viewpoint. The study was conducted at Muscat College from January to March 2024.Simple randomization implemented at the course level helped to choose the participants. All students registered in the specified courses were asked to take part after a random sample of the courses Muscat College offers throughout the study term was chosen. Five hundred fifty students completed the survey. AMOS was used to conduct confirmatory factor analysis (CFA), and PSS was used to evaluate the internal consistency ( IBM Corp., 2024a, 2024b).

**Results:**

CFA of the SEEQ indicated that the eight -factor solution best fit the data. Results also showed that the SEEQ has overall good-to-excellent reliability. The adapted scale was psychometrically sound, proving SEEQ’s applicability

**Conclusions:**

This study proposes using SEEQ to measure Oman’s education quality.

## 1. Introduction

Course and program evaluation is a critical quality control process performed by all colleges and universities. Student evaluations of teaching (SET), typically administered through questionnaires with rating scales (e.g., Likert scales), enable students to express their degree of agreement or disagreement with various statements. Initially designed for evaluating courses and programs, SETs have expanded to assess teaching effectiveness ( Marsh, 1987). Marsh (1987) defines teaching effectiveness as the assessment of various instructional components, including clarity of presentation, instructor enthusiasm, organization, interaction with students, and feedback provision. These factors reflect pedagogical skills beyond the subject matter and demonstrate how SETs assess both course quality and teaching effectiveness.

One widely used tool in educational research is the Student Evaluation of Educational Quality (SEEQ) developed by Marsh (1982). When developing SEEQ, several aspects of teaching and learning were taken into account, enabling its wide-ranging use in a variety of academic fields. SEEQ is globally recognized as a reliable and effective instrument for measuring teaching effectiveness ( Balam & Shannon, 2010; Coffey & Gibbs, 2001; Grammatikopoulos et al., 2015). Strong internal consistency has been demonstrated by SEEQ’s psychometric qualities; in a variety of international situations, Cronbach’s alpha scores range from 0.88 to 0.97 ( Marsh, 1982; Marsh & Roche, 1993). For example, a study in Oman by Al-Hinai (2011) reported a Cronbach’s alpha of 0.87, affirming its applicability within the Omani context. Despite these positive results, the SEEQ has yet to be fully explored within Oman’s higher education system.

However, Constantinou and Wijnen-Meijer (2022) emphasize the importance of critically acknowledging SETs’ shortcomings. They point out that SETs are subject to potential biases such as gender bias, response bias, and the influence of non-teaching characteristics such as course difficulty or instructor popularity, which can affect assessments of teaching effectiveness. These biases may affect the validity of SETs as a solitary measure of instructional quality. As a result, while SETs provide valuable data, they should be utilized cautiously and in conjunction with other evaluation methodologies to ensure a more comprehensive and equitable assessment of teaching quality.

Although SETs and tools like SEEQ are useful in the context of Oman’s higher education system, more research is required to examine their validity and reliability in a variety of cultural and educational contexts. By examining the psychometric qualities of the SEEQ among Omani college students, this study seeks to close this gap. The objectives of this study are to: (a) assess the Arabic version of SEEQ’s reliability and (b) use confirmatory factor analysis to determine its dimensional structure.

## 2. Methods

### 2.1 Study design

The Students’ Evaluations of Educational Quality (SEEQ) Questionnaire ( Marsh & Roche, 1993) was utilized in this quantitative study to measure educational quality from the students’ perspective. A quantitative approach was chosen for its effectiveness in gathering numerical data that can be statistically analyzed to uncover correlations and patterns within the dataset ( Creswell & Creswell, 2017). This method provides an objective, measurable evaluation of educational quality, aligning with the research objectives. Although qualitative methods like interviews or case studies could offer deep insights into teaching effectiveness, a quantitative approach was preferred for its ability to generalize findings across a large sample and for its statistical rigor ( Creswell & Creswell, 2017). The study took place at Muscat College from January to March 2024. The findings aim to contribute to the broader discussion on evaluating higher education by collecting data on student assessments of course quality ( Hancock & Algozzine, 2016).

### 2.2 Sample size calculation and sampling method

A total of 278 students needed to be surveyed to achieve a 95% confidence interval, a 5% significance level, and 80% power. The participants were selected by a simple randomization process. The participants were chosen through a simple randomization process at the course level. Completion is based upon checking the registration department for a comprehensive list of Muscat College students.

Simple randomization implemented at the course level helped to choose the participants. Muscat College was chosen because it is the researcher’s workplace, making it convenient for distributing the questionnaire. Furthermore, the college’s wide array of departments, such as computing, business, and foundation courses, offered an ideal environment for collecting diverse student responses. The selection process was seeking to guarantee a wide representation of courses from different academic disciplines (e.g., computing, business, and foundation), course sizes (small to large lecture-based classrooms), and academic years (first year to senior-level students). All students enrolled in randomly selected classes were invited to participate. This approach was designed to capture a diverse and representative sample of student experiences, giving a solid foundation for assessing the findings’ generalizability. Five hundred fifty students completed the survey. The response rate was 91.7%, with 550 out of 600 individuals participating.

### 2.3 Participants

In the current study, 550 students from three different departments at Muscat College participated by completing the questionnaires (SEEQ) distributed to them. The participants averaged 20 years old, with a 2.54-year standard deviation.

### 2.4 Ethical approval

The ethical approval was received by Muscat College’s Research Ethical Committee under the reference MC/RC/1/2024, dated January 2024. Prior authorization was obtained before starting the search. Because the study only required completing a non-invasive, anonymous survey (SEEQ) measuring student assessments of educational quality, which had been approved by the research committee, all participants provided oral permission before they were contacted. Participants were told that their participation in this research was founded on the principles of confidentiality and autonomy, and that they might withdraw from the study at any moment.

### 2.5 Data collection procedures

The Students’ Evaluations of Educational Quality (SEEQ) Questionnaire was used for data collecting; the chosen sample of students received paper-based questionnaires. The scheduled hour for the questionnaire administration was set; the teacher stayed outside the classroom to provide an objective environment while students were completing the surveys. Following completion of the surveys, a chosen student collected them, sealed them in an envelope, and handed the envelope to the authors. Students were advised that the teacher would not get any direct comments from the assessments, therefore ensuring the privacy of their answers. Three months, from January 2024 to March 2024, defined the data collecting period. Following the ethical standards set by the college, all completed surveys are accompanied by signed informed permission forms.

To maintain anonymity, all data were securely stored, and no identifiable information was recorded. The study followed the college’s ethical guidelines, with informed consent obtained from all participants. Data collection occurred from January to March 2024.

### 2.6 Measures

The Students’ Evaluations of Educational Quality (SEEQ) questionnaire was the tool used ( Marsh, 1982). The SEEQ consists of thirty items that measure eight dimensions under consideration, one of which measures the subject’s overall evaluation. The SEEQ consists of the following dimensions: breadth of coverage, assessment, assignments, learning value, teacher enthusiasm, organization, group interaction, individual rapport, and overall rating ( Marsh, 1982). Each component is rated on a five-point Likert scale: strongly disagree, disagree, neutral, agree, and strongly agree ( Marsh, 1982). The tool’s developer, Professor Herbert March, was contacted personally and granted permission to be used for study ( Marsh, 1982). The author translated SEEQ into Arabic prior to the study, and the instrument was then translated back into English by a bilingual expert who has experience in this area.

Discrepancies that were identified were rectified in the Omani version of the instrument after the bilingual expert and the authors conducted a comparison between the two editions of the instrument (the original and the re-translated version). Subsequently, a preliminary test was administered to a selected group of students and colleagues to evaluate the reliability of the translated SEEQ. Modifications were implemented to improve the semantic significance of some words in Arabic.

### 2.7 Data analysis

The SEEQ scale was validated using a combination of alternate methods and data analysis. An evaluation of the internal consistency of the scale and its three subscales was conducted using Cronbach’s alpha statistics. The structure of the SEEQ factors was examined using confirmatory factor analyses (CFAs) in AMOS v26, which is a structural equation modeling program. The model was evaluated using fit indices that are often used in the discipline ( Blunch, 2008). The Tucker-Lewis index (TLI), goodness-of-fit index (GFI), comparative fit index (CFI), and Chi-Square (χ ^2^) are among the measures. The model fit indices were deemed acceptable if the Comparative Fit Index (CFI), Goodness of Fit Index (GFI), and Tucker-Lewis Index (TLI) were above 0.90, and the Root Mean Square Error of Approximation (RMSEA) was below 0.08 ( Bentler, 1990).

## 3. Results

### 3.1 Reliability of internal consistency

To determine the internal consistency reliability of each SEEQ scale, Cronbach’s alpha was used. The output was assessed using the corrected item-total correlation or alpha if an item was omitted ( Field, 2013). The reliability (alpha) coefficients for the eight 30-item SEEQ scales are presented in Table 1.

**Table 1.** Cronbach’s Alpha for the scale.

Subscale	No of items	Alpha
learning value	4	0.72
Enthusiasm	4	.77
Organization	4	.72
Group interaction	4	.76 .76
Individual rapport	4	.76
BREADTH	4	.76 .65
Examination	3	.65
ASSIGNMENTS	2	.68
Overall Cronbach’s Alpha for the scale	30	.95

### 3.2 Confirmatory factor analysis

**Figure 1.**
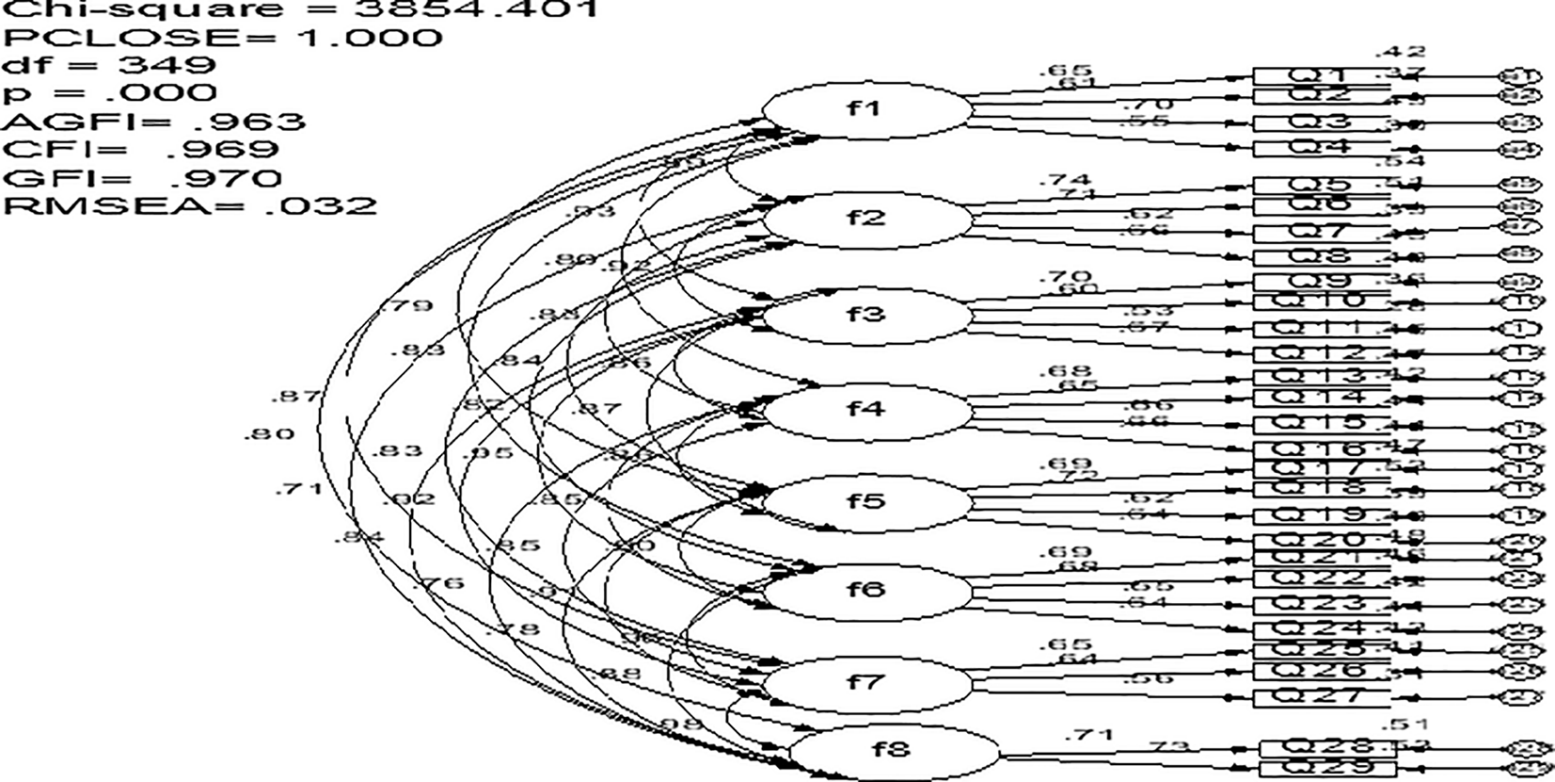
The measurement model for SEEQ.

Confirmatory factor analysis (CFA) is the most suitable technique for cross validating the factor structure of a test, as per Byrne (2001). Using the AMOS version 26.0 statistical tool, a covariance matrix ( Figure 1) was used to evaluate the validity of the item allocation to the eight SEEQ measures ( IBM Corp, 2024a). AMOS v26 was selected due to its sophisticated modeling capabilities and user-friendly interface, which make it well-suited for managing complex structural equation models. The eight-factor structure proposed for the original scale has been confirmed by the Arabic version of SEEQ’s acceptable CFA indices ( Figure 1).

## 4. Discussion

The revised CFA model’s goodness-of-fit indices indicate a strong fit to the study data: χ ^2^ (df=349)=3854.401, CFI=.963, GFI=.970, PCOLOSE=1.00, and RMSEA=.032. These indices all exceeded thresholds. Cronbach’s Alpha showed 0.935 reliability, demonstrating high internal consistency. However, applying Cronbach’s alpha and Confirmatory Factor Analysis (CFA) in a different cultural and linguistic context, such as the Arabic version of SEEQ in Oman, may present challenges. These challenges include potential variations in how scale items are interpreted due to language nuances and cultural differences that could affect students’ perceptions and responses. These factors should be considered when generalizing findings across different populations. The findings indicate that SEEQ is a valuable tool for evaluating both educational programs and individual courses in Oman’s higher education system. While this study demonstrates its applicability, further research with larger and more diverse samples is necessary to enhance the generalizability of the results to the broader student population in Oman. Despite the current sample limitations, SEEQ remains useful for program-level evaluations aimed at improving overall educational quality.

### Ethical approval

The ethical approval was received by Muscat College’s Research Ethical Committee under the reference MC/RC/1/2024, dated January 2024. Prior authorization was obtained before starting the search. The nature of the research and the little risk involved led to oral consent instead than written permission. Participants in the research had to complete a non-invasive, anonymous survey (Students’ Evaluations of Educational Quality - SEEQ), which only gauges their opinions of educational quality. Written consent was believed to be not required given the low-risk nature of the survey and its anonymous form. The research committee authorized the oral consent procedure as well as the SEEQ tool. All participants provided oral permission before they were contacted. Participants were told that their participation in this research was founded on the principles of confidentiality and autonomy, and that they might withdraw from the study at any moment.

### Consent statement

All participants provided informed consent before participating in the study. The consent process was approved by the college’s ethics committee. Participants agreed that their data would be used for research purposes and could be published. They also agreed to the anonymous use of their data, with no personal information published and the data presented without distortion of scientific meaning.

I declare that the data provided is true and can be accounted for.

## Data Availability

Figshare: Data 1_1 - Copy.xls https://doi.org/10.6084/m9.figshare.26494288.v2 ( Al-kalbani, 2024a). This project contains the following underlying data:
• Data 1_1 - Copy.xls Data are available under the terms of the Creative Commons Zero (CC0) All data has been deidentified using the Safe Harbour approach, therefore assuring ethical norms’ conformance. Among the authors who helped to produce this dataset was Muna Alkalbani. Figshare: SEEQ-Arabic version.pdf https://doi.org/10.6084/m9.figshare.27222894.v5 ( Al-kalbani, 2024b). Additionally, the translated SEEQ questionnaire, the ethical approval for this research, and the informed consent template are available upon request. For more information, please contact the corresponding author. This project contains the following extended:
• ilovepdf_merged (3).pdf Data are available under the terms of the Creative Commons Zero (CC0)

## Software availability statement

IBM SPSS Statistics and IBM AMOS, proprietary program created by IBM Corp., were used in the data analysis for this research. Similar statistical analysis and structural equation modeling may be done using free substitutes such PSPP and the R package lavaan. Lavaan may be accessible via the R environment at https://lavaan.ugent.be/. PSPP is available at [ https://www.gnu.org/software/pspp/].
