# Supplementary material for: Assessing the students’ evaluations of educational quality (SEEQ) questionnaire in Oman higher education
Source: F1000Res. 2025 Feb 10;13:1329. Originally published 2024 Nov 6. [Version 2] doi: 10.12688/f1000research.157354.2 (PMC12171714; doi:10.12688/f1000research.157354.2)
Supplement: Peer review history

# Reviewer report: Reviewer response for version 2

**DOI:** 10.5256/f1000research.177084.r387744

**Published:** 2025-06-16

**Authors:** Suranto Suranto

**Affiliations:** 1 Universitas Muhammadiyah Surakarta, Surakarta, Central Java, Indonesia

1. The background section of this article has presented the fundamental theories regarding the importance of teaching evaluation and the position of SEEQ as an internationally recognized instrument. The references used are quite strong, including Marsh’s research and several other relevant studies. To strengthen the contextual foundation, the author should consider adding recent studies from countries in the Middle East or other developing nations that have conducted validations of the SET/SEEQ instrument. In addition, the research gap needs to be emphasized more explicitly — for example, by stating that to date, no study has comprehensively tested the structural validity and reliability of the Arabic version of SEEQ using CFA in the context of Oman.

 2. The use of a quantitative research method with CFA as the primary analytical tool is appropriate for testing the construct validity and reliability of the instrument. The explanation regarding the sampling procedure and the use of AMOS for analysis is also clear. However, the author should provide more detailed information about the back-translation process, including who the translators were, their qualifications, as well as the mechanism of the preliminary instrument testing (number of participants, implementation procedures, and a brief summary of the results). In addition, it is necessary to explain the reason for adopting all 30 items in the SEEQ without modification, or if any modifications were made, which parts were adjusted. Information about the total student population at Muscat College should also be included so that readers can understand the proportion of the sample relative to the overall population.

 3. The presentation of the instrument’s reliability results through Cronbach’s alpha values is very good and demonstrates high internal consistency across most subscales. It would be even better if the author provided the CFA results not only in the form of goodness-of-fit indices but also with a table of factor loadings for each SEEQ item. Additionally, the discussion of the reliability and validity results should be further elaborated by explaining which factors contributed the most or the least, and which factors were most responsive to the Omani context.

 4. The discussion has addressed important aspects such as the implications of applying the instrument in different cultural contexts and the challenges of language adaptation. However, the discussion would be stronger if it also included theoretical implications for the development of cross-cultural validity studies of teaching evaluation instruments. In addition, the practical implications for higher education institutions in Oman, particularly regarding teaching quality policies and faculty evaluation systems, should be explained more comprehensively. The study’s limitations should also be described in greater detail — for example, regarding the research subjects being limited to a single institution and the absence of external validity testing.

 5. The conclusion has effectively summarized the research findings and stated that the SEEQ is suitable for use in higher education in Oman. However, it would be stronger if it included a recommendation for implementing the SEEQ at the institutional level as a tool for monitoring the quality of teaching and learning. Additionally, suggestions for future research should be made more specific — for example, by recommending invariance testing across semesters, institutions, or academic programs to ensure the stability of the factors in various contexts.

Is the work clearly and accurately presented and does it cite the current literature?

Partly

If applicable, is the statistical analysis and its interpretation appropriate?

Partly

Are all the source data underlying the results available to ensure full reproducibility?

Yes

Is the study design appropriate and is the work technically sound?

Yes

Are the conclusions drawn adequately supported by the results?

Partly

Are sufficient details of methods and analysis provided to allow replication by others?

Partly

Reviewer Expertise:

I specialize in the field of educational management, which focuses on quality assurance, evaluation systems, and the development of educational policies within institutional settings. Research on the validation of the SEEQ instrument is highly relevant because teaching evaluation tools are a crucial component of educational quality management systems, particularly to ensure that student evaluations of lecturers are conducted using valid, reliable, and contextually appropriate measurement instruments.

I confirm that I have read this submission and believe that I have an appropriate level of expertise to confirm that it is of an acceptable scientific standard, however I have significant reservations, as outlined above.

# Reviewer report: Reviewer response for version 2

**DOI:** 10.5256/f1000research.177084.r365522

**Published:** 2025-02-12

**Authors:** Dickson Okoree Mireku

**Affiliations:** 1 University of Cape Coast, Cape Coast, Ghana

Everything is fine. Please go ahead and accept the manuscript for indexing. Thanks

Is the work clearly and accurately presented and does it cite the current literature?

No

If applicable, is the statistical analysis and its interpretation appropriate?

No

Are all the source data underlying the results available to ensure full reproducibility?

No

Is the study design appropriate and is the work technically sound?

No

Are the conclusions drawn adequately supported by the results?

No

Are sufficient details of methods and analysis provided to allow replication by others?

No

Reviewer Expertise:

NA

I confirm that I have read this submission and believe that I have an appropriate level of expertise to confirm that it is of an acceptable scientific standard.

# Reviewer report: Reviewer response for version 1

**DOI:** 10.5256/f1000research.172790.r347907

**Published:** 2025-01-02

**Authors:** Dickson Okoree Mireku

**Affiliations:** 1 University of Cape Coast, Cape Coast, Ghana

1.    Clarity and Accuracy of Presentation and Citation of Current Literature

 The introduction does not reference relevant literature, citing only a few sources such as Al-Muslim and Arifin (2015) and Constantinou and Wijnen-Meijer (2022). This is because authors failed to adequately connect the literature about students appraisal or evaluation of teaching performances (presumably what the authors aimed to review) about the context of higher education in Oman. This limitation will affect the discussion of the work as the authors may not have the opportunity to thoroughly discuss the findings of the study in relation to the reviewed literature. In this regard, the literature review needs to be more extensive to strengthen the conceptual and theoretical foundation of the work.

 To enhance the study, the following literature areas should be reviewed:

 a)    The role of student evaluations in assessing teaching effectiveness and educational quality.

 b)    Cross-cultural validation and adaptation of psychometric tools in higher education.

 c)    Reliability and validity of the SEEQ questionnaire across different contexts.

 d)    Best practices for using student evaluations to improve instructional methods.

 e)    Challenges and implications of implementing SETs in higher education institutions in the Middle East.

 There is a lack of seamless transition between the global applications of SEEQ and its specific implementation in Oman, resulting in a disjointed narrative.

 Many cited studies are relatively old, and incorporating more recent literature could enhance the relevance of the discussion.

 2.    Study Design Appropriateness and Academic Merit

 a)    While a quantitative approach is employed, the justification for selecting this methodology over alternatives for evaluating the psychometric properties of SEEQ is not provided.

 b)    The sampling method is outlined, but it does not clarify the rationale behind choosing Muscat College and its three departments, limiting the broader relevance of the results.

 c)    Although ethical considerations are acknowledged, the write-up lacks specifics on how anonymity and data protection were maintained beyond simply obtaining oral consent.

 3.    Adequate Details of Methods and Analysis for Replication

 a)    The explanation of the questionnaire administration process is lacking, as it does not clarify how discrepancies in the translated instrument were resolved beyond basic semantic adjustments.

 b)    The approach for calculating sample size lacks thorough justification for the selected parameters, including the 95% confidence interval and 80% power.

 c)    While confirmatory factor analysis (CFA) is mentioned, there is insufficient detail on how the model's fit indices were computed and interpreted.

 3. Appropriateness of Statistical Analysis and Interpretation

 a)    Although Cronbach’s alpha and CFA are utilized effectively, the write-up does not critically examine the potential limitations of these methods for cross-validating SEEQ in a different cultural and linguistic context.

 b)    There is no rationale provided for choosing AMOS v26, nor is there a discussion on its advantages over alternative software.

 c)    While the discussion section highlights the findings positively, it fails to adequately consider the biases that may arise from the small sample size and the study's focus on a single institution.

 4. Availability of Source Data and Reproducibility

 a)    The write-up fails to specify how others can access the translated SEEQ questionnaire or the raw data, hindering transparency and reproducibility.

 b)    While the process for securing permission from the tool’s developer is briefly mentioned, it lacks information on how the adapted Arabic version can be shared for future research.

 c)    There is no indication of whether the complete ethical approval document or informed consent template is available for review, which would enhance reproducibility.

 5. Suggestions about the general outlook of the paper/study:

 A)    More recent and regionally relevant studies should be incorporated to strengthen the introduction and discussion sections.

 B)    A justification for methodological choices would benefit from a clearer focus on context-specific relevance and limitations.

 C)    Additionally, more detailed explanations regarding sample size calculation, ethical considerations, and translation processes are warranted.

 D)    A critical assessment of the limitations of the statistical tools used and the contextual nuances in their interpretation would be beneficial.

 E)    Enhancing transparency by specifying where supplementary materials (e.g., the dataset, translated questionnaire) can be accessed is also recommended.

Is the work clearly and accurately presented and does it cite the current literature?

No

If applicable, is the statistical analysis and its interpretation appropriate?

No

Are all the source data underlying the results available to ensure full reproducibility?

No

Is the study design appropriate and is the work technically sound?

No

Are the conclusions drawn adequately supported by the results?

No

Are sufficient details of methods and analysis provided to allow replication by others?

No

Reviewer Expertise:

NA

I confirm that I have read this submission and believe that I have an appropriate level of expertise to state that I do not consider it to be of an acceptable scientific standard, for reasons outlined above.

# Response

**Published:** 2025-01-15

**Authors:** Muna Al Kalbani

**Affiliations:** Muscat College, Ruwi, Muscat Governorate, Oman

Thank you for your insightful feedback. In response to your first comment regarding the literature review’s scope and relevance, I have significantly revised the introduction and literature review to address the areas you highlighted:

1. I expanded the discussion on the **role of student evaluations** in teaching quality assessment, incorporating more recent literature.
2. I included additional studies on the **cross-cultural adaptation** of psychometric tools and the **reliability and validity** of the SEEQ in various contexts.
3. The **transition between global and local applications** of SEEQ was clarified, highlighting the need for further validation in Oman.
4. I added **more recent and regionally relevant sources** to enhance the timeliness of the discussion.
  These revisions strengthen the theoretical framework and align the findings more closely with existing research.

In response to your second comment:

1. I added a **justification for the quantitative methodology**, emphasizing its suitability for statistical analysis and generalizing results (Creswell & Creswell, 2017).
2. I provided a **rationale for selecting Muscat College**, explaining how its diverse disciplines, course sizes, and academic years offer a representative sample.
3. I elaborated on **ethical considerations**, detailing data protection measures, secure storage, and the non-collection of identifiable information.

Also, I revised the methods section to include:

1. A **detailed explanation of the translation process**, specifying how discrepancies were addressed.
2. A **justification for the sample size parameters**, referencing standard guidelines for confidence intervals and statistical power.
3. Additional information on **CFA fit indices**, specifying thresholds for CFI, GFI, TLI, and RMSEA (Bentler, 1990).

additionally, In response to your third comment:

1. I added a discussion on the **limitations of Cronbach’s alpha and CFA** for cross-cultural validation, highlighting challenges with language nuances and cultural differences.
2. I provided a **rationale for selecting AMOS v26**, discussing its advantages over alternatives.
3. I acknowledged potential **biases related to sample size** and the single-institution focus, emphasizing this as a limitation.

In response to your fourth comment:

1. I clarified that **the translated SEEQ questionnaire and raw data** will be available upon request.
2. I included **information on sharing the adapted version** for future research.
3. I noted the **availability of ethical approval documents and consent templates** for review.

In response to your suggestions, I have made the following revisions:

1. I incorporated **recent and regionally relevant studies** to strengthen the introduction and discussion.
2. I provided **clearer justifications for methodological choices**.
3. I elaborated on **sample size, ethical considerations, and translation**.
4. I added a **critical assessment of statistical tool limitations**, focusing on cultural context.
5. I clarified **access to supplementary materials**, including the dataset and translated questionnaire, to enhance transparency.

# Reviewer report: Reviewer response for version 1

**DOI:** 10.5256/f1000research.172790.r344928

**Published:** 2024-12-20

**Authors:** Margret Sigrun Sigurdardottir

**Affiliations:** 1 Faculty of Business Administration, University of Iceland, Sæmundargata, Iceland

Thank you for the opportunity to review the article *Assessing the students’ evaluations of educational quality (SEEQ) questionnaire in Oman higher education. *The article discusses the use of Student Evaluation of Teaching (SET) tools, particularly the SEEQ, to assess teaching effectiveness and evaluate courses and programmes. While the topic is significant and timely, the authors fall short in addressing critical issues surrounding SET's broader use and the nuances involved in its application.

 The authors claim that while SET was originally designed to evaluate courses and programmes, it has since been repurposed to assess teaching effectiveness. However, this statement is presented without any supporting references. It would strengthen the article to provide evidence explaining how a tool intended for course evaluation has been adapted to measure teaching effectiveness and the mechanisms through which this occurs.

 A related point concerns the citation of Constantinou & Wijnen-Meijer (2022). While the paper is referenced to substantiate the claim that SET evaluates various aspects of teaching, the authors neglect to engage with the controversial aspects raised in the same work. For example, there is no discussion of the criticism surrounding SET, such as its potential biases or limited reliability in assessing teaching quality. This omission becomes particularly pronounced when the authors assert that SET has been used to support valuable decisions in academic career development—a statement that is neither referenced nor critically examined.

 The authors state that SEEQ is widely used worldwide and is regarded as one of the most accurate and useful tools for measuring teaching effectiveness. However, this sweeping assertion is supported by a single citation that discusses the SEEQ's usability in evaluating quality within Arabic secondary education in Malaysia. The reliance on this narrow context does not justify claims of global applicability or general reliability. A more nuanced review of SEEQ's strengths and limitations across varied educational settings would provide greater validity to this argument.

 The methods section lacks sufficient detail on the selection of courses evaluated using the SEEQ. For instance, the authors do not provide information on the diversity of subjects, course sizes, or academic years represented in the sample—all of which could significantly influence the SET results. Including such details topic would enable readers to assess the generalisability and robustness of the findings.

 Additionally, the discussion on SEEQ’s application to programmes versus individual courses is inconsistent. The authors emphasise SEEQ's use in evaluating education programmes but later recommend its application for individual instructors seeking feedback on modules. This raises questions about the scope and intended purpose of the SEEQ in the context of Oman’s higher education system. The need for further research is also highlighted, but the rationale for this call is unclear. Specifically, the authors suggest that more varied samples are needed to generalise findings to Omani students, yet they simultaneously endorse SEEQ's utility for evaluating programmes. This contradiction requires clarification.

 The consent statement asserts that participants gave informed oral consent to be interviewed. However, the article does not present any interview data, which renders this inclusion confusing. Further, the sentence describing participant consent—“The source of the informants was who agreed that the data were provided for research purpose and that the data can be published…”—is poorly written and lacks coherence. It should be rephrased for clarity and accuracy.

 The references list exhibits inconsistencies, with some citations appearing twice and formatting varying between bolded and highlighted titles. Ensuring adherence to a consistent citation style would improve the article’s professional presentation.

Is the work clearly and accurately presented and does it cite the current literature?

Partly

If applicable, is the statistical analysis and its interpretation appropriate?

Partly

Are all the source data underlying the results available to ensure full reproducibility?

Partly

Is the study design appropriate and is the work technically sound?

Partly

Are the conclusions drawn adequately supported by the results?

Partly

Are sufficient details of methods and analysis provided to allow replication by others?

Partly

Reviewer Expertise:

Higher Education teaching and learning, Scholarship of teaching and learning, qualitative research,

I confirm that I have read this submission and believe that I have an appropriate level of expertise to confirm that it is of an acceptable scientific standard, however I have significant reservations, as outlined above.

# Response

**Published:** 2025-01-15

**Authors:** Muna Al Kalbani

**Affiliations:** Muscat College, Ruwi, Muscat Governorate, Oman

Thank you for your insightful feedback. In response to your first comment regarding the lack of evidence supporting the adaptation of Student Evaluations of Teaching (SETs) from course and programme evaluation to teaching effectiveness assessment, I have revised the manuscript to include additional references. Specifically, I have added a citation to Marsh (1987), which explains the mechanisms by which SETs assess various teaching dimensions, including clarity, organization, instructor enthusiasm, and student interaction. This provides a stronger foundation for the claim and addresses the broader use of SETs.

 Whereas, in response to your second comment concerning the citation of Constantinou & Wijnen-Meijer (2022) and the omission of a critical discussion on SET’s potential biases and reliability issues, I have revised the manuscript accordingly. I engaged with the controversies highlighted by the authors, particularly regarding biases and the limitations in measuring teaching quality. This addition enhances the discussion's depth and ensures a more balanced and nuanced perspective.

 In response to your third comment about the overgeneralization of SEEQ's global applicability, I have included additional references to support the claim. The revised manuscript now provides a more balanced discussion of SEEQ’s strengths and limitations in diverse educational contexts, reinforcing its broader applicability with stronger, more varied evidence.

 In response to your fourth comment about the lack of methodological detail, I have revised the Methods section to describe the diversity of subjects, course sizes, and academic years included in the sample. Additionally, I clarified the randomization process used for course selection. These enhancements improve the context and allow a more robust assessment of generalisability.

 Additionally, in response to your fifth comment regarding the inconsistency between SEEQ’s application for programmes and individual courses, I have clarified the discussion to state explicitly that SEEQ is applicable to both contexts. The revised manuscript resolves the contradiction by highlighting the need for larger and more diverse samples to enhance generalisability while maintaining SEEQ’s utility for various evaluation purposes.

 Also, in response to your sixth comment, I have removed the reference to interviews in the consent statement since no interview data were collected. The consent description has also been rephrased for clarity and coherence, accurately reflecting the approved consent process by the ethics committee. Finally, in response to your final comment regarding reference inconsistencies, I have thoroughly reviewed the references and corrected duplicate entries and formatting issues. The reference list now adheres to a consistent citation style, enhancing the article's overall professionalism and readability.
